# Molybdenum Diboride (MoB_2_) Nanoparticles via a Facile Molten Salt Route: Synthesis, Characterization, Cytotoxicity, and Antibacterial Studies

**DOI:** 10.1021/acsabm.5c00161

**Published:** 2025-07-14

**Authors:** Hamide Aydın, Burcu Üstün, Utkan Şahintürk, Nurdan Sena Değirmenci, Fikrettin Şahin, Sedef Kaptan Usul, Ayşe Aslan, Muslum Demir, Ümran Kurtan

**Affiliations:** † Department of Chemistry, İstanbul University-Cerrahpaşa, Istanbul 34500, Türkiye; ‡ Materials Institute, The Scientific and Technological Research Council of Türkiye (TUBITAK) − Marmara Research Center (MAM), Gebze 41470, Türkiye; § Department of Chemical Engineering, İstanbul University-Cerrahpaşa, Istanbul 34500, Türkiye; ∥ Department of Mechanical and Metal TechnologiesVocational School of Technical ScienceZ, Istanbul University-Cerrahpaşa, Istanbul 34500, Türkiye; ⊥ Institute of Nanotechnology and Biotechnology, Istanbul University-Cerrahpaşa, Istanbul 34500, Türkiye; # Department of Genetics and Bioengineering, Faculty of Engineering, Yeditepe University, Istanbul 34755, Türkiye; ∇ Bioengineering Department, 52962 Gebze Technical University, Kocaeli 41400, Türkiye; ○ Gebze Technical University, Institute of Energy Technologies, Kocaeli 41400, Türkiye; ◆ Department of Chemical Engineering, Boğaziçi University, Engineering Faculty, Istanbul 34342, Türkiye; ¶ Department of Materials and Materials Processing Technologies Vocational School of Technical Sciences, Istanbul University-Cerrahpaşa, istanbul 34500, Türkiye

**Keywords:** molybdenum diboride (MoB_2_), molten salt, cytotoxicity on cancer, antibacterial activity

## Abstract

Herein, nanocrystalline MoB_2_ powder synthesized via a single-step molten salt reaction of precursor materials MoCl_5_ and amorphous boron powder in the presence of KCl and NaCl by varying the boron amount was applied for the first time in vitro cytotoxicity and antibacterial studies. The crystalline structure, morphology, and surface characteristics were investigated in detail by powder X-ray diffraction (XRD), X-ray photoelectron spectroscopy (XPS), scanning electron microscopy (SEM), high-resolution transmission electron microscopy (HRTEM), and Brunauer–Emmett–Teller (BET) analysis. The results indicated that the amount of boron played a crucial role in the nanostructure of MoB_2_. XRD results showed an enhancement in crystallinity, and HRTEM examinations revealed a rise in reflections (100) and growth in grains with the rise in boron amount during the synthesis of MoB_2_. The particle size was found to be in the range of 50–100 nm, and the surface areas of MoB_2_ nanoparticles were measured between 7.74 and 16.11 m^2^/g. In vitro findings showed that MoB_2_-16 did not have a significant cytotoxic effect on healthy cells (HaCaT and MCF10A), but it exhibited a notable cytotoxic effect on breast cancer cell lines (MDA-MB-231 and MCF7 cells), whereas no cytotoxic effect occurred on the liver cancer line Hep3B cells. Antibacterial studies revealed that MoB_2_-4 exhibited promising antibacterial activity against both and , where MICs were in the range of 60 to 70 μg/mL. Overall, our research reported the successful synthesis and characterization of MoB_2_ nanoparticles, which can be efficient anticancer and antibacterial agents.

## Graphical abstract

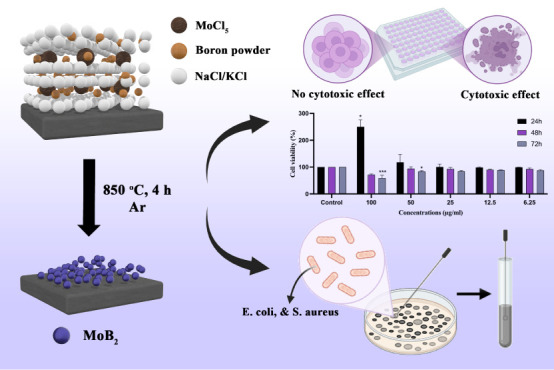

## 1 Introduction

Nanomaterials (NMs) display unique physical, chemical, and biological characteristics resulting from their nanoscale size and significantly increased surface area compared to bulk materials. Various metal-based nanostructured materials have been studied for their functions in medicine, biotechnology, environment, and energy storage.^1–3^ NMs are classified into two main categories: inorganic and organic NMs. Inorganic NMs include metallic NPs (Zn, Mo, and Mn), semiconductor NMs (CdS and ZnO), and magnetic NMs (ZnFe_3_O_4_, SnF_2_O_4_), while organic NMs comprise carbon nanotubes, carbon nanofibers, and polymer-based NPs. Among inorganic NMs, molybdenum-based (Mo-based) nanomaterials such as molybdenum sulfide (MoS_2_), molybdenum carbide, molybdenum phosphides, and molybdenum oxide have recently garnered significant interest due to their unique physicochemical features.^4,5^

On the other hand, molybdenum borides offer advantages such as low cost, ease of synthesis, chemical stability, structural diversity, and the potential to serve as substitutes for precious metal catalysts, and they are especially attractive due to their structural diversity.^6^ The strong M–B and highly covalent B–B bonds display various compositions and crystal structures such as Mo_2_B, α-MoB, β-MoB, and MoB_2_.^7,8^ Boron, with its intermediate electronegativity of 2.04 on the Pauling scale, facilitates the formation of diverse bond types, including ionic bonds (M–B), covalent bonds (B–B), and metallic bonds (M–M/M–B).^9^ They exhibit exceptional mechanical, chemical, and catalytic properties, structural complexity, thermal durability, and superconductivity.^10^

Molybdenum borides can be synthesized through a range of chemical and physical techniques, including chemical vapor deposition (CVD),^11^ physical vapor deposition (PVD),^12^ selective etching methods,^13^ and molten salt-assisted methods.^14^ These methods have their advantages and disadvantages. Among nanomaterial synthesis methods, the molten salt technique is regarded as a modified form of the solid-state reaction (SSR), in which a molten salt acts as a solvent to dissolve solid reactants and solvate ions through strong polarization and facilitates the rapid movement of reactant species via transfer and diffusion. Molten salts aid in the production of pure products with uniform and controlled size and shape at relatively lower temperatures compared with the SSR method. It stands out as a distinctive approach and is promising, environmentally friendly, and cost-effective.^15,16^ The molten salt technique also offers several advantages, such as simplicity, efficient product formation, large-scale production, low cost, and environmental friendliness.^17,18^ Nanomaterials synthesized via this method typically exhibit high homogeneity, a purer phase, and controlled size and morphology. These attributes make them well-suited for a wide range of applications such as energy storage,^19^ hydrogen storage,^20^ and biological fields such as cancer treatment and antibacterial applications.

Cancer prevalence and mortality rates are rising globally, making it a leading cause of death. “The Global Cancer Statistics 2020” report records approximately 19 million new cancer cases globally in 2020.^21^ The primary cancer treatment methods, including surgery, chemotherapy, and radiotherapy, have limited effectiveness. Recently, as a biological application, nanomaterials offer unique physical properties and surface chemistry, allowing for the regulation of cellular behavior. These nanomaterials designed for cancer have recently had greater effects than traditional therapeutic agents.^22^ Molybdenum is a found dietary element that is vital for the human body. Several molybdenum-dependent enzymes, such as aldehyde oxidase, xanthine oxidase, and sulfite oxidase, are involved in critical metabolic functions within the organism.^23^ Investigations have shown the potential of Mo-based heterocomposites, such as molybdenum dichalcogenides, molybdenum oxides, and Mo-based polyoxometalates, in the treatment and detection of various cancers, including breast,^24^ lung,^21^ liver, and gastric cancer.^25^ Their chemical stability, biocompatibility, circulation properties, and targeting capabilities make them promising candidates for cancer therapies.^22,26^ In cancer treatments, molybdenum disulfide has been shown in vitro studies to increase the proliferation of human dermal fibroblast cells (HDFs), human umbilical cord endothelial cells (HUVECs), and human hair dermal papilla cells (HhDPCs), which are important for wound healing.^27^ On the other hand, bacterial infections are considered a major threat to human health. The emergence of bacterial resistance, driven by the increasing need for antibiotics and their irrational use, represents a critical global challenge. The increasing problem of antibiotic resistance has driven a shift in research toward exploring alternative antibacterial strategies beyond traditional antibiotics.^28,29^ In recent years, metal and metal-oxide-based nanoparticles have attracted increasing attention in the biomedical field due to their antimicrobial properties.^30^ They can overcome antibiotic resistance by killing bacteria through mechanisms such as cell wall damage, reactive oxygen species (ROS) generation, and interactions with proteins and DNA.^31^ Mo-based nanomaterials have attracted significant attention in antibacterial research due to their unique physicochemical properties. These properties primarily exert their effects through mechanisms such as oxidative stress, the generation of ROS, and the binding of nanomaterials to intracellular components, leading to direct damage to bacterial cell walls and membranes.^32,33^ Additionally, the low toxicity of Mo-based nanomaterials enhances their appeal as an attractive material for antibacterial applications.^34^ Boron and its derivatives are prominent among inorganic antibacterial agents, offering significant potential for antimicrobial and biocompatible applications.^35^ These compounds directly target microbial cell membranes, causing structural damage and generating oxidative stress, which can ultimately lead to cell death.^36^ In addition to their established antibacterial effects, boron and its derivatives are also known to interfere with protein synthesis and disrupt the function of specific enzymes in microorganisms when used in high concentrations.^37–40^ In the literature, it is reported that molybdenum oxide (MoO_*x*_) and molybdenum sulfide (MoS_2_) nanoparticles show high antibacterial activities.^32,41^ Therefore, molybdenum diboride (MoB_2_) could be a promising material and is relatively underexplored in terms of its biological potential, particularly in the context of its cytotoxicity against cancer cells and its antibacterial properties.

Herein, this study aims to synthesize molybdenum diboride depending on boron concentration through the efficient and easy method of molten salt, characterize these borides using XRD, SEM, HRTEM, XPS, and BET, and first investigate their anticancer and antibacterial activities. The findings of this research could pave the way for the development of other boride-based nanomaterials for anticancer and antibacterial applications.

## 2 Materials and Methods

### 2.1 Materials

Molybdenum pentachloride (MoCl_5_, Sigma-Aldrich, 99%) and nanoboron (amorphous powder, Solarbio, ≥95%) were used as precursors, while sodium chloride (Merck, 95%) and potassium chloride (Alfa Aesar, 95%) salts were used as molten salts. Yeast extract powder, agar powder, and peptone were purchased from Sigma-Aldrich.

### 2.2 Experimental Section

MoB_2_ nanoparticles were prepared via a single-step molten salt reaction. In this process, MoCl_5_ and boron powder were combined in the molar ratio of Mo:B (1:4–8–16) and mixed with 2.5 g of NaCl-KCl (45:55 by weight) salt in an agate mortar for 5 min. The mixture was then heated to 850 °C at 8 °C/min under an argon atmosphere and kept at this temperature for 4 h. Then, the mixture was allowed to cool naturally to room temperature. After solidification, the salts were removed by washing with DI water, and the boride particles were dried at 60 °C under vacuum overnight (Scheme 1).

**Scheme 1:**
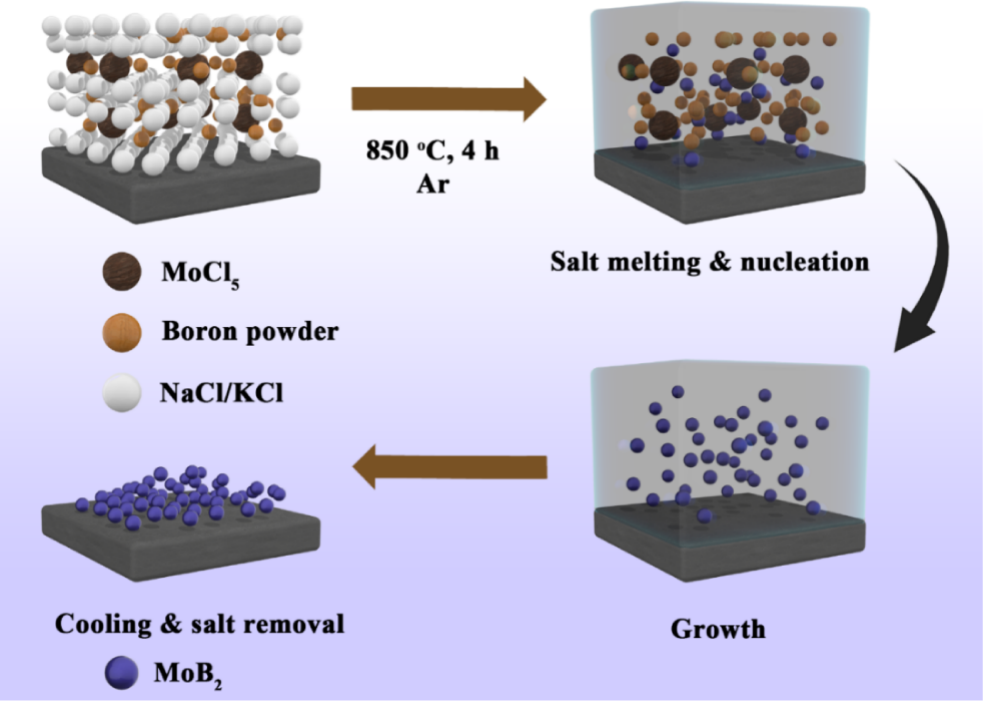
Schematic Illustration of the Synthesis of MoB_2_ Nanoparticles

### 2.3 Cell Culture

MoB_2_-16, MoB_2_-8, and MoB_2_-4 were prepared at a 1 mg/mL concentration in a complete cell medium and solubilized via vortexing. In this work, we used a human skin cell line called HaCaT (ATCC HB-241), a human breast cell line called MCF 10A (CRL-10317, ATCC), a hepatocellular carcinoma cell line called Hep3B (HB-8065, ATCC), and two breast cancer cell lines, MCF-7 (HTB-22, ATCC) and MDA-MB-231 (HTB-26, ATCC). We cultivated HaCaT, Hep3B, MCF-7, and MDA-MB-231 cells in DMEM high-glucose medium (Gibco 11965092, Thermo Fisher Scientific, USA), supplemented with 10% fetal bovine serum (FBS, Gibco 10500–056, Thermo Fisher Scientific, USA) and 1% Penicillin/Streptomycin/Amphotericin (PSA, Invitrogen, Gibco, UK).

MCF 10A cells were cultured in the MEBM Bullet kit (2.00 mL of BPE, 0.50 mL of hEGF, 0.50 mL of insulin, 0.50 mL of hydrocortisone) (Lonza CC-3150, Switzerland), supplemented with 10% FBS and 1% PSA. All cells were grown in a humidified incubator at 37 °C with 95% air and 5% CO_2_. Once the cells reached 70% confluence in the tissue culture flask (TPP, Z707503, Merck, Germany), the culture medium was discarded, and they were carefully washed with phosphate-buffered solution (PBS) (Euroclone, Italy) before proceeding to further culture and treatment protocols.

### 2.4 Cell Viability Assay

HaCaT, MCF 10A, MCF 7, MDA-MB-231, and Hep3B cells were seeded onto 96-well plates (TPP, Switzerland) at a density of 5000 cells/well. Subsequently, several concentrations of MoB_2_-16, MoB_2_-8, and MoB_2_-4 (100, 50, 25, 12.5, and 6.25 μg/mL) were prepared in the media. After 24 h, HaCaT was treated with the stated concentrations of MoB_2_-16, MoB_2_-8, and MoB_2_-4; MCF 10A, MCF 7, MDA-MB-231, and Hep3B cells were treated with the stated concentrations of MoB_2_-16 for 24, 48, and 72 h. Cell viability was measured using the MTS reagent (G3582, CellTiter 96 Aqueous One Solution; Promega, Southampton, UK) according to the manufacturer’s instructions. Absorbance at 490 nm was detected using an ELISA plate reader (Biotek, Winooski, VT). The percentage of cell viability was calculated according to the control cells (nontreated for MoB_2_). All studies were performed in triplicate.

### 2.5 Antibacterial Studies

The antibacterial activity of the synthesized MoB_2_ nanoparticles was assessed by using minimum inhibitory concentration (MIC) measurements. MIC testing was conducted against Gram-negative (ATCC 25150) and Gram-positive (ATCC 6538) bacterial strains, following the methodology described by Kaptan Usul et al.^42^ MoB_2_ concentrations ranging from 50 to 150 μg/mL, prepared in DMSO (dimethyl sulfoxide), were applied to the plates to determine the inhibitor concentrations. As controls, bacterial suspensions in the LB medium (Lysogeny broth) without the material and in the medium containing only DMSO were tested. We incubated the plates at 37 °C for 24 h. After incubation, the bacterial viability was assessed by observing the clearance of turbidity. The lowest concentration at which no visible bacterial growth was observed for each MoB_2_ sample was recorded as the MIC value. Following the MIC measurements of MoB_2_ particles, minimum bactericidal concentration (MBC) testing was conducted for the bacterial strains. MBC has the lowest particle concentration at which no visible growth appears on the LB agar plates. For MBC determination, 3 μL of bacterial culture from the wells showing no visible growth in the MIC microtiter plate after 18 h of incubation was inoculated onto agar plates.^43^ All experiments were conducted with a minimum of three replicates for each concentration to ensure reliability and precision.

## 3 Result and Discussion

### 3.1 Characterization of MoB_2_ Samples

The crystallinity and phase characteristics of the synthesized samples were analyzed by using XRD. Figure 1 shows that the positions and intensities of the main peaks are in good agreement with the typical peaks of hexagonal MoB_2_ (space group *P*6/*mmm*, COD (Crystallographic Open Database) Card No. 96–151–0764). The diffraction angles of 2θ = 29.59°, 34.63°, 45.70°, 60.76°, 61.75°, 69.85°, 71.64°, 79.50°, 40.67°, and 46.06° correspond to the (001), (100), (101), (002), (110), (111), (200), and (210) crystal planes of MoB_2_, respectively. It is worth noting that the diffraction peaks at 2θ = 60.77° and 61.96° of MoB_2_-8 were seen as clearer at 60.76° and 61.75° in the sample of MoB_2_-16, indicating the better crystallinity feature of MoB_2_-16. All diffraction angles of 2θ degrees for other samples are also shown in Table S1. The samples’ average crystallite sizes were 8.49, 9.02, and 7.22 nm for MoB_2_-4, MoB_2_-8, and MoB_2_-16 calculated by the Debye–Scherrer’s equation, respectively.^44^ The interlayer distances between crystal planes *d*_001_, *d*_100_, and *d*_101_ were obtained using the Bragg formula, and the values are shown in Table 2.^45^ As a result, XRD analysis demonstrated the highly crystalline nature of the synthesized samples.

**Figure 1:**
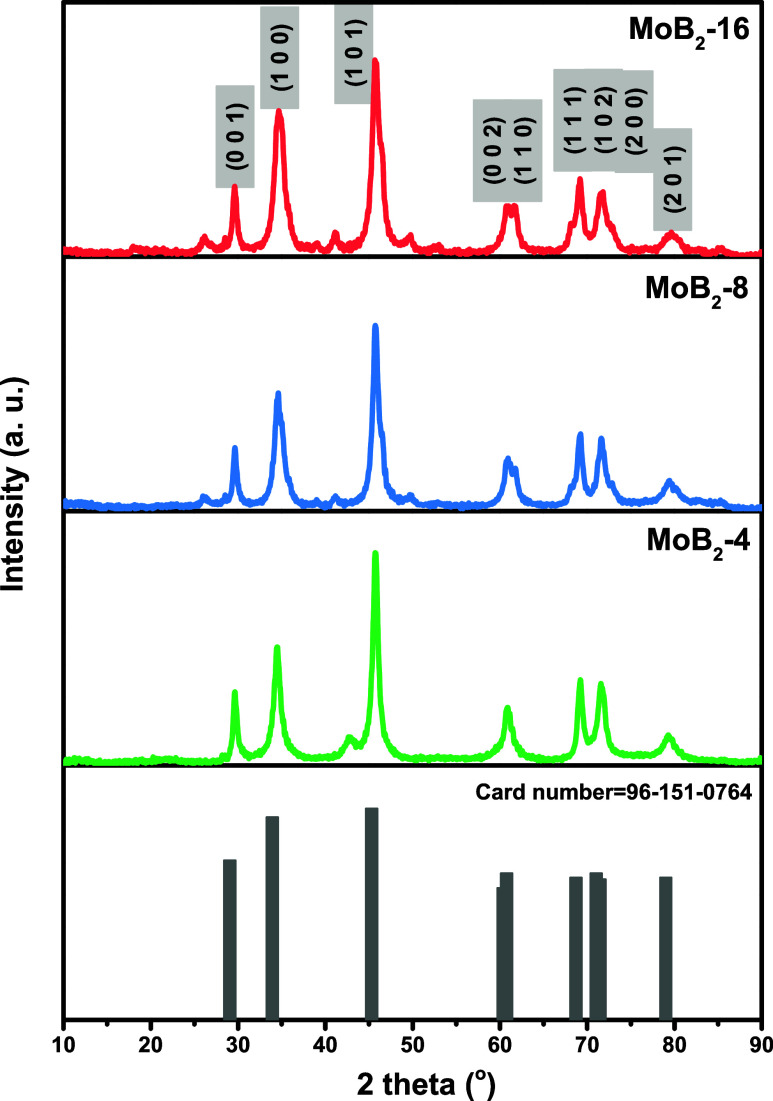
XRD patterns of the synthesized MoB_2_ samples compared with the theoretical pattern of MoB_2_ (bottom).

XPS examination was performed to further analyze the synthesized MoB_2_-8 sample. The survey spectrum consisted of 187.1 eV B 1s, 233.1 eV Mo 3d, 284.1 eV C 1s, and 531.1 eV O 1s (Figure S1). Common C 1s and O 1s impurities were also present in molybdenum boride systems, in addition to the molybdenum and boron peaks, which were typical characteristics often found in the XPS survey of metal borides.^46^ As can be seen in Figure 2a, four oxidation states of Mo^0^, Mo^3+^, Mo^4+^, and Mo^6+^ were found by deconvolution of the Mo 3d XPS spectra. Mo^0^ (226.78 and 231.28 eV) corresponded to the molybdenum of molybdenum boride, while various molybdenum oxides (228.08 eV, 233.48 eV, 230.18 eV, and 234.88 eV) corresponded to the surface oxidation of MoB_2_.^47,48^ The deconvoluted peaks in the B 1s spectrum indicated the presence of two distinct boron atoms. The higher binding energy value at 187.5 eV confirmed the B^0^ state of metal boride (Figure 2b),^46,49^ while the lower binding energy at 186.1 eV typically confirmed the B–B bond of elemental boron.^50,51^ In particular, the absence of any boron oxide phase suggested that the boron on the surface was not oxidized.^52^ Peak positions and atomic percentages are listed in Table 1. All these results were compatible with those obtained from previously described molybdenum boride materials in the literature.^47,48^

**Figure 2:**
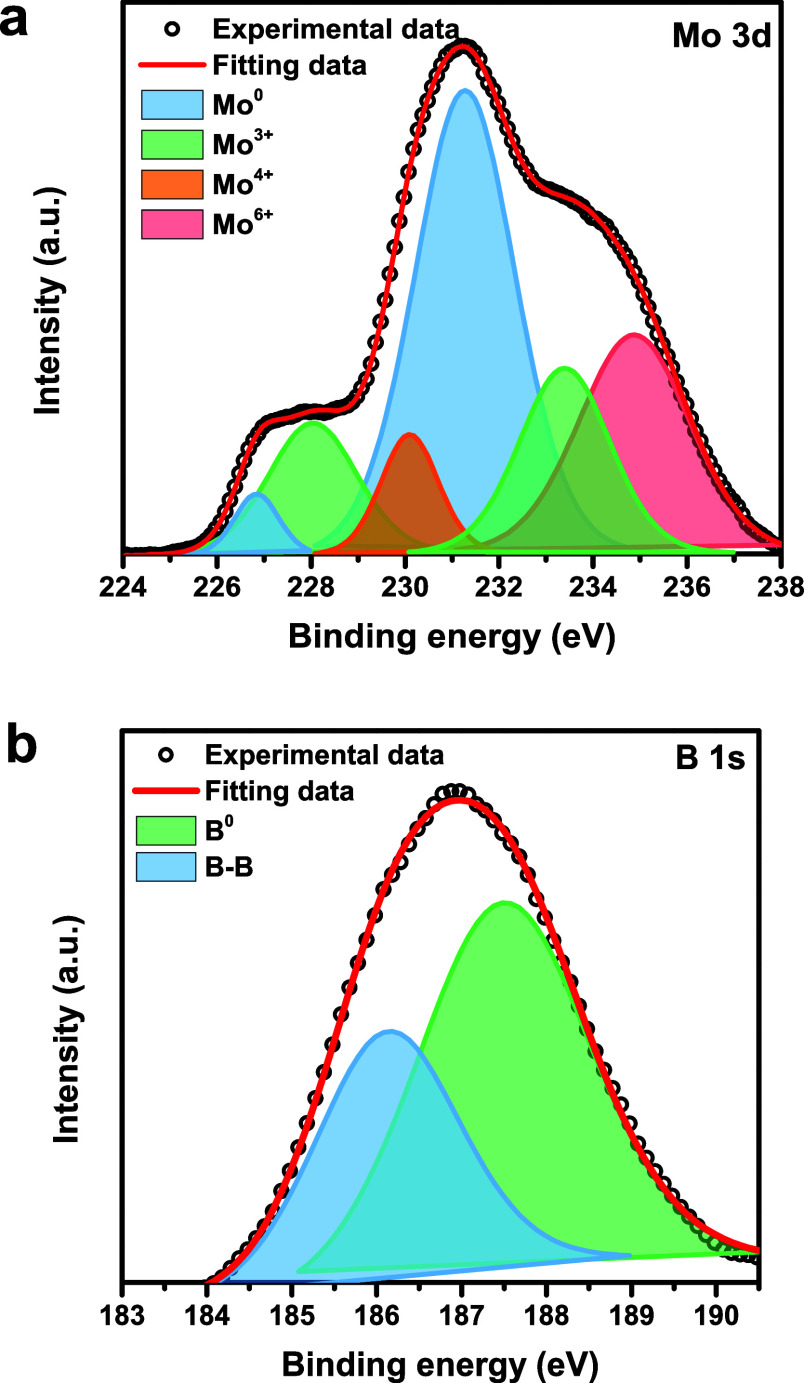
Deconvoluted XPS spectra of MoB_2_-8, for (a) Mo 3d and (b) B 1s.

**Table 1:** Chemical Composition of MoB_2_-8

MoB_2_–8	Species	Peak position for Mo 3d/B 1s (eV)	At. %
Mo 3d			75.4
MoB_2_	Mo^0^	226.78; 231.28	32.42
Mo-oxide	Mo^3+^	228.08; 233.48	28.93
	Mo^4+^	230.18	19.41
	Mo^6+^	234.88	19.25
B 1s			24.6
MoB_2_	B^0^	187.5	57.27
B–B	B^0^	186.1	45.73

The SEM micrographs presented in Figure 3a–c show the morphological features of MoB_2_-4, MoB_2_-8, and MoB_2_-16 samples, respectively. It has been observed that the amount of boron used significantly influences the morphology of MoB_2_ nanoparticles. The key feature of porous transition metal borides is the formation of porous networks through the aggregation of nanoscale building blocks like nanoparticles.^53^ Therefore, the overlapping created an agglomerated morphology, which can be caused by the uneven distribution of MoB_2_ nanoparticles and may be associated with mesoporosity. The particle size was almost in the range of 50–100 nm.

**Figure 3:**
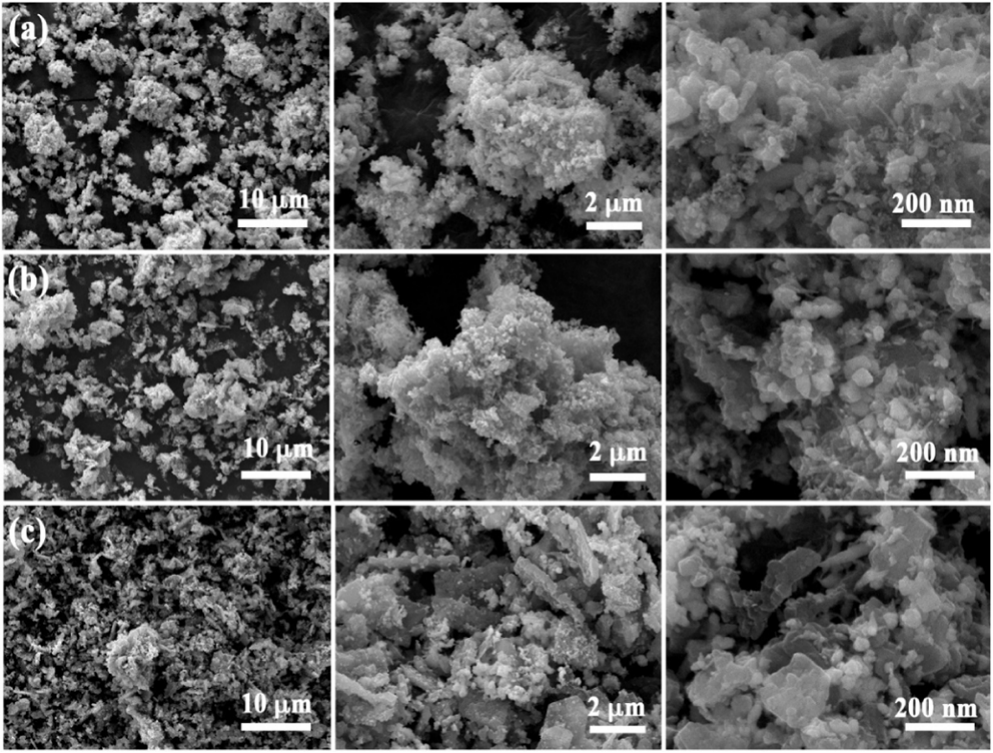
SEM images of (a) MoB_2_-4, (b) MoB_2_-8, and (c) MoB_2_-16.

Figure 4a–c shows the TEM analysis results of MoB_2_-4, MoB_2_-8, and MoB_2_-16, and as can be seen from the micrographs, a spherical-like morphology was observed. Figure S2 shows that the corresponding energy-dispersive spectroscopy (EDS) mappings confirm the uniform distributions of Mo and B elements. HRTEM images show atomic planes, grain boundaries, and crystal defects in the MoB_2_ nanostructure (Figure 4d–f). The orientation of crystallites can be described as a random texture for MoB_2_-4. It was seen that the grain orientation was significantly affected by the change in the boron amount during the synthesis of MoB_2_.^54^ Grain growth and the (100) orientation plane of the samples increased significantly from MoB_2_-4 to MoB_2_-16. The lattice spacing with the peaks of the first three most intense planes (001), (100), and (101) of MoB_2_ can be observed in the selected area electron diffraction (SAED) pattern images in Figure 4g–i. The typical HRTEM image of MoB_2_-8 as shown in Figure 4h discerns lattice fringes with interplanar spacings of 0.31, 0.263, and 0.206 nm, corresponding to the (001), (100), and (101) planes of MoB_2_-8, respectively. The calculated *d*-values from XRD were compatible with the measured values (Table 2). The clear spots corresponding to the (001), (100), and (101) planes from SAED further confirm the presence of a crystalline structure in the molybdenum boride, and this was also in agreement with the XRD results (Table 2).

**Figure 4:**
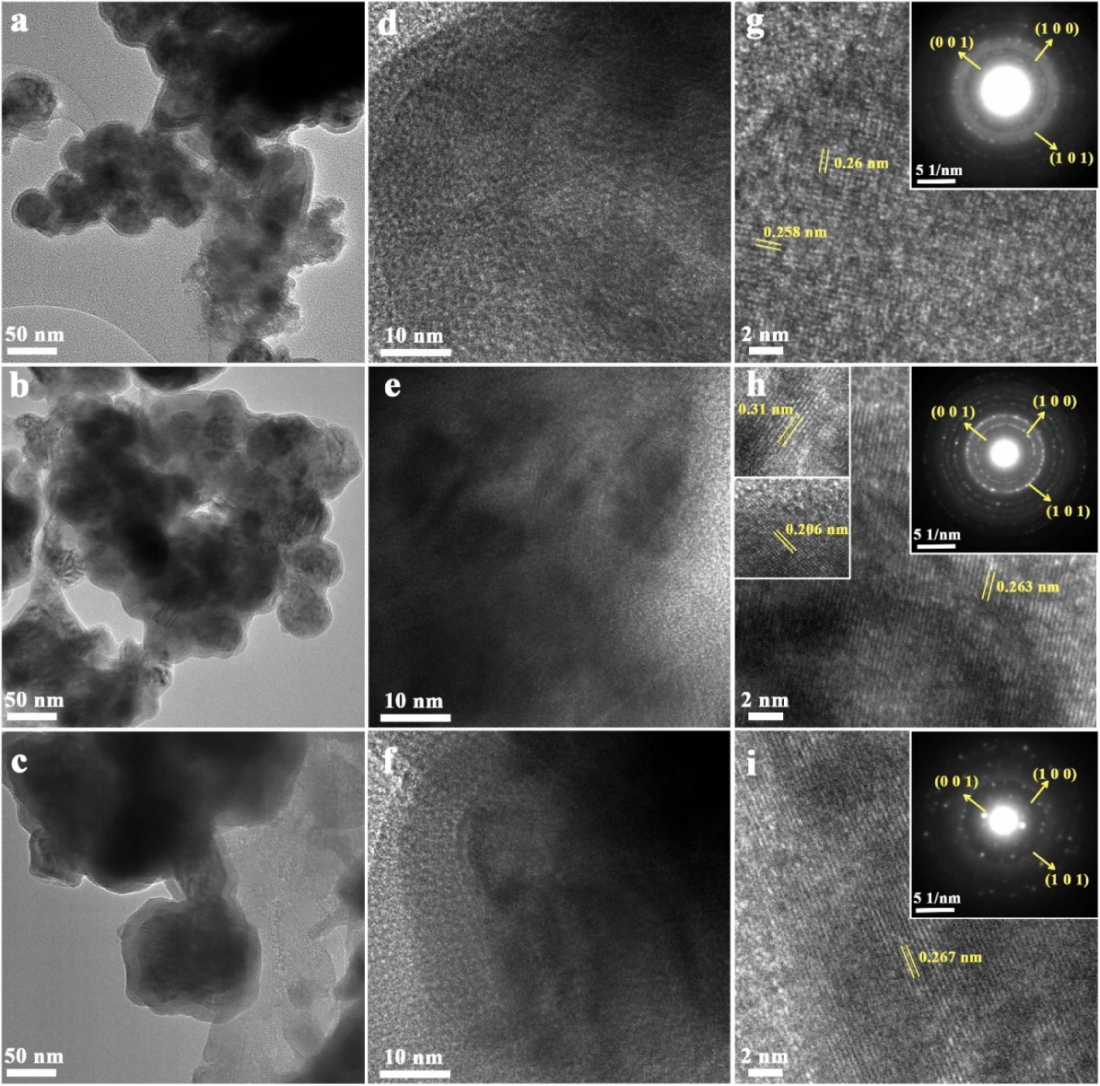
(a–c) TEM images, (d–f) HRTEM, and (g–i) SAED pattern images of MoB_2_-4, MoB_2_-8, and MoB_2_-16, respectively (top-to-down).

**Table 2:** Calculated and Measured Interplanar Spacing (*D*) Values from XRD and HRTEM, Respectively

Calculated *d*-values from XRD (nm)						Measured *d*-values from HRTEM (nm)		
***h***	***k***	***l***	MoB_2_-4	MoB_2_-8	MoB_2_-16	MoB_2_-4	MoB_2_-8	MoB_2_-16
0	0	1	0.301	0.300	0.302	-	0.310	-
1	0	0	0.260	0.259	0.259	0.260–0.258	0.263	0.267
1	0	1	0.198	0.198	0.198	-	0.206	-

Figure 5a,b shows that N_2_ adsorption–desorption experiments were used to assess the BET surface area and the pore characteristics of the three specimens. MoB_2_-4 possessed the highest surface area, followed by MoB_2_-8 and MoB_2_-16. It was clear that the surface area and mesoporosity changed with the amount of boron used in the synthesis of molybdenum borides. The lowest surface area of 7.74 m^2^/g of MoB_2_-16 may suggest a significant degree of aggregation due to the excess amount of boron. Additionally, crystalline species generally exhibit a lower surface area than amorphous species, which arises from the structural organization of the materials.^55^ According to XRD and HRTEM results, MoB_2_-16 was the most crystalline species and possessed the lowest surface area. The obtained surface areas (7.74–16.11 m^2^/g) were not seen as large values, but they were approximately 2–4 times greater than the bulk MoB_2_ (4.23 m^2^/g) fabricated by arc melting.^48^ Table 3 shows the BET surface area values and other pore characteristics.

**Figure 5:**
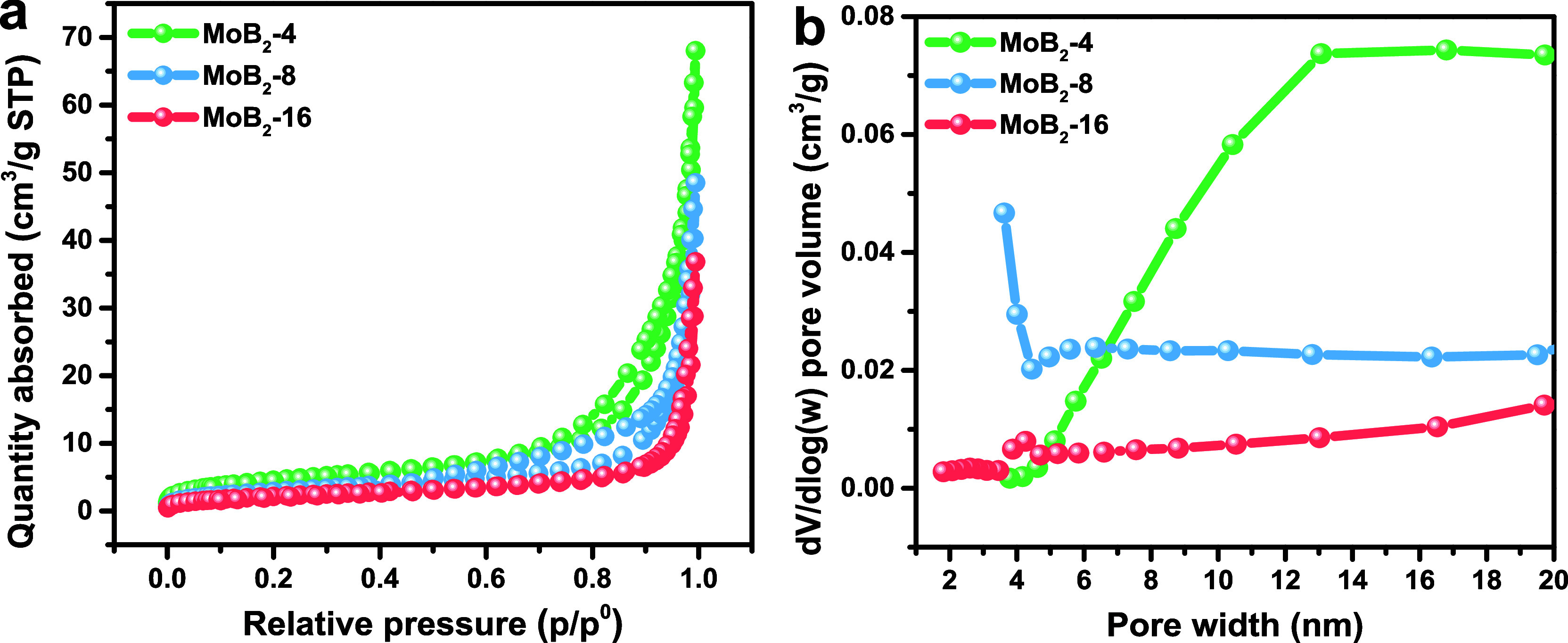
(a) BET isotherm and (b) PSD of the MoB_2_ samples.

**Table 3:** *S*_BET_, and Pore Characteristics of the MoB_2_ Samples

Samples	*S*_BET_ (m^2^/g)	*V*_tot_ (cc/g)	*V*_mic_ (cc/g)	*V*_meso_ (cc/g)	*D* (nm)
MoB_2_-4	16.11	0.078	0.00187	0.07613	24.33
MoB_2_-8	10.38	0.049	0.00207	0.04693	21.62
MoB_2_-16	7.74	0.033	0.00142	0.03158	30.16

### 3.2 Cytotoxic Effects of MoB_2_ on HaCaT, MCF 10A, MCF 7, MDA-MB-231, and Hep3B Cell Lines

Molybdenum-containing compounds, such as molybdenum disulfide (MoS_2_), are of interest in the biomedical field due to their biocompatibility in biological systems. The literature has demonstrated the cytotoxic effect of the molybdenum sulfides in the combination study on the MDA-MB-231 cell line.^56^ Literature studies have also shown the efficacy of molybdenum oxide (MoO_3_), a molybdenum molecule, in cancer therapy. A study has demonstrated the cytotoxic effect of MoO_3_ on skin cancer cells.^57^

In this research, we investigated the cell viability and the cytotoxic effect of MoB_2_-16 in HaCaT, MCF 10A, MCF 7, MDA-MB-231, and Hep3B cells using the MTS cell proliferation assay over 24, 48, and 72 h. Cell viability was assessed as a percentage relative to that of the control cells. HaCaT cells were treated with MoB_2_-4 and MoB_2_-8, as well as MoB_2_-16. As shown in Figure S3, 100 μg of MoB_2_-4 and MoB_2_-8 treatments significantly reduced cell viability in HaCaT cells, especially at 48 and 72 h. Therefore, we examined the treatment effects of MoB_2_-16 on cancer cells (MCF 7, MDA-MB-23, and Hep3B cells) and healthy breast cells (MCF10 A). From Figure 6, the cell viability at 24 h in all cells treated with 100 μg of MoB_2_ was increased compared to the control group. Furthermore, at 24, 48, and 72 h, the cells of HaCaT (Figure 6a) and MCF10 A (Figure 6b) (the healthy cell groups) treated with 6.25, 12.5, 25, 50, and 100 μg of MoB_2_-16 showed no cytotoxic effects. In MCF 7 (Figure 6c) and MDA-MB-231 (Figure 6d) cells treated with 100 μg of MoB_2_, cell viability was significantly reduced at 48 and 72 h. Additionally, we observed the absence of harmful effects in Hep3B (Figure 6e) cells exposed to MoB_2_-16.

**Figure 6:**
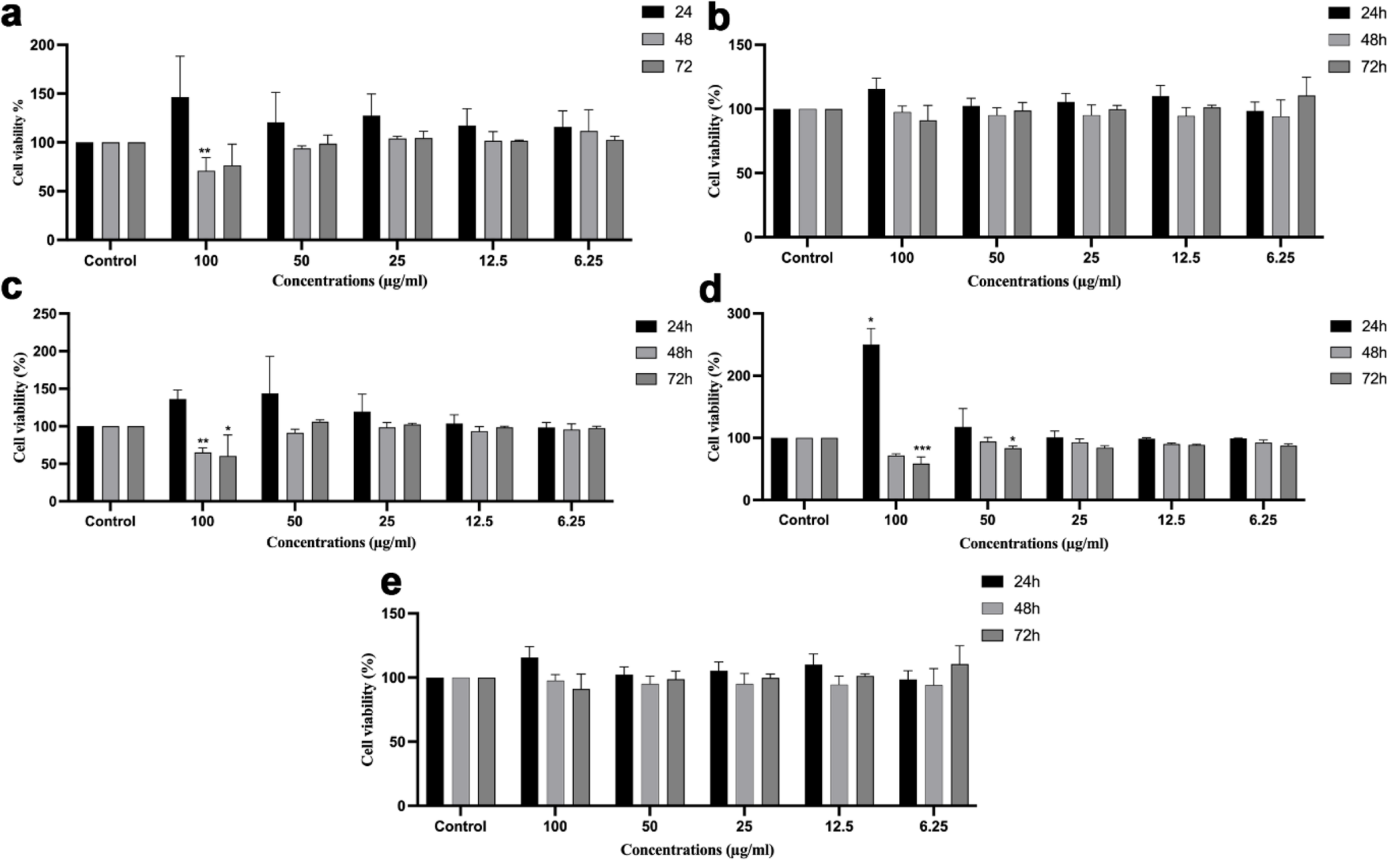
Effect of MoB_2_-16 treatment on the viability of HaCaT, MCF 10A, MCF 7, MDA-MB-231, and Hep3B cell lines. 6.25–100 μg of MoB was treated on (a) HaCaT, (b) MCF10 A, (c) MCF 7, and (d) MDA-MB-231, and (e) the MTS assay was conducted on Hep3B cells at 24, 48, and 72 h, with measurements taken on each consecutive day of treatment. Absorbance was measured at 490 nm using a microplate reader. The data represent the average of three independent experiments ± SD (***p ≤ 0.001, **p ≤ 0.01, and *p ≤ 0.05).

This study’s results indicate that MoB_2_-16 exhibited greater activity on HaCaT cells due to its higher boron content compared to the MoB_2_-4 and MoB_2_-8 nanoparticles. Increasing the boron amount facilitates an increase in the therapeutic effect within the cell. Recent investigations have demonstrated the potential of various boron derivative combinations as anticancer agents for hepatocellular carcinoma.^58,59^ Consistent with our prior investigations, our research also indicates that boron enhances the efficacy of molybdenum and increases its stability for cells.

### 3.3 Antibacterial Results of MoB_2_

Studies on boron-containing materials have demonstrated their inherent antibacterial properties.^34,60,61^ However, the antibacterial activity of MoB_2_ nanoparticles has not been previously investigated. In this study, the antibacterial efficacy of MoB_2_ nanoparticles was assessed against and through minimum inhibitory concentration (MIC) and minimum bactericidal concentration (MBC) measurements, as summarized in Table 4. The MBC results for these bacterial strains are illustrated in Figure 7. Gram-positive bacterial cell walls were characterized by a thick peptidoglycan layer and a high concentration of teichoic acid, whereas Gram-negative bacteria possessed a thinner peptidoglycan layer associated with an outer membrane composed of lipopolysaccharides and phospholipids.^35,36,42^ Despite these distinct structural differences, MoB_2_ nanoparticles were observed to penetrate bacterial cell walls and exhibit antibacterial activity against both Gram-positive and Gram-negative . Among the tested materials, MoB_2_-4 demonstrated the highest antibacterial activity against both and , with MIC values ranging from 60 to 70 μg/mL. The antibacterial activity of MoB_2_-8 and MoB_2_-16 nanoparticles was comparatively lower. Specifically, MoB_2_-8 exhibited MIC and MBC values of 90–100 μg/mL and 100–110 μg/mL, respectively, against . The MIC and MBC values for MoB_2_-16 particles were consistent for both and , falling within the range of 110–120 μg/mL. Nanomaterials exhibit diverse mechanisms of antibacterial activity, which are strongly influenced by their physicochemical properties, including size, shape, and surface area.^62^ The enhanced efficacy against both Gram-negative and Gram-positive is due to the larger surface area of the MoB_2_-4 nanomaterial, as determined by BET analysis. Specifically, the extensive surface area of MoB_2_-4 facilitates direct interaction with bacterial cells and allows it to penetrate cell membranes or walls, causing membrane disruption and resulting in the leakage of intracellular contents.^33^ Furthermore, oxidative stress and electron transfer between MoB_2_-4 and bacterial cells can also play a crucial role in the antibacterial mechanism.^63^

**Table 4:** MIC and MBC Values (μg/mL) for and

Bacteria	Particles	MIC* (μg/mL)	MBC* (μg/mL)
	MoB_2_-4	60–70	60–70
	MoB_2_-8	90–100	100–110
	MoB_2_-16	110–120	110–120
	MoB_2_-4	60–70	70–80
	MoB_2_-8	110–120	110–120
	MoB_2_-16	110–120	110–120

**Figure 7:**
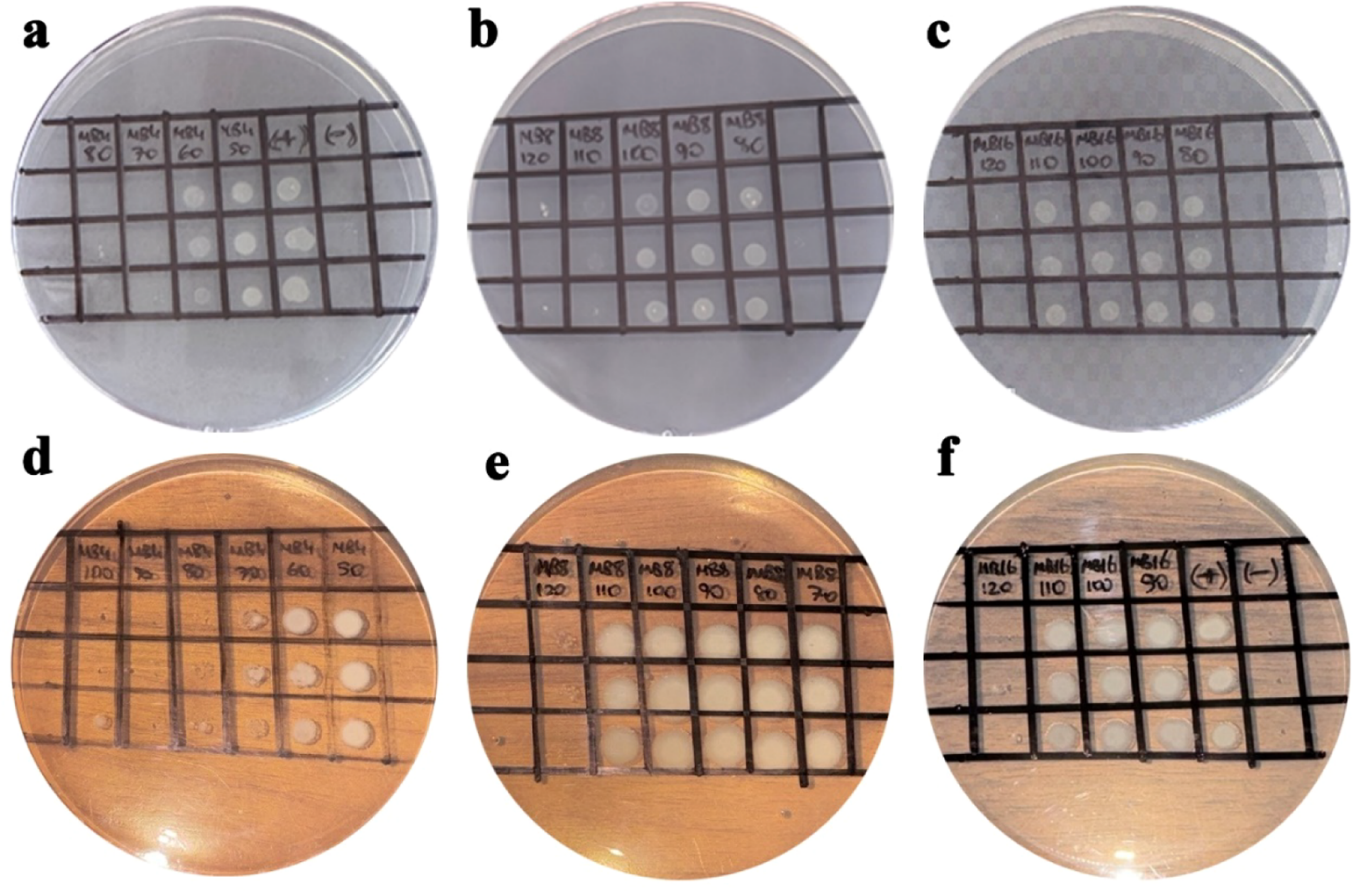
MBC images against (a–c) and (d–f) of MoB_2_-4, MoB_2_-8, and MoB_2_-16, respectively (left-to-right).

## 4 Conclusions

In conclusion, the molten salt method offers a reliable and scalable approach to the synthesis of MoB_2_ nanoparticles. XRD analysis revealed the highly crystalline nature of the MoB_2_ nanoparticles with a size range of 50–100 nm. MoB_2_-16 possessed the lowest surface area, and according to the cytotoxicity results, MoB_2_-16 did not show a significant cytotoxic impact on healthy cells (HaCaT and MCF-10A), even at elevated dosages. This finding indicated that MoB_2_-16 may serve as a safe treatment for healthy cells. The treatment of the hepatoma cell line Hep3B with MoB_2_-16 showed no cytotoxic effect. However, treatment of breast cancer cell lines MCF7 and MDA-MB-231 with 100 μg of MoB_2_-16 led to a significant reduction in cell viability. This discovery suggests that a specific dosage of MoB_2_-16 is effective in certain cancer cell lines, and MoB_2_-16 could function as an anticancer agent against breast cancer cells, especially the triple-negative type. Additionally, among the tested samples, MoB_2_-4 nanoparticles exhibited higher efficacy against both Gram-positive and Gram-negative bacterial cells due to their higher surface area, and their MIC values were in the range of 60 to 70 μg/mL. With their unique properties and promising biological activities, MoB_2_ nanoparticles have the potential to serve as new materials for a wide range of biomedical applications, including anticancer therapy and infection control.

## Supplementary Material



## Notes

Additional experimental details, microscopic images, XRD patterns data, XPS spectra, TEM elemental mapping, and treatment on the viability of HaCaT cell line (PDF)

## Abbreviations

%: percentage
μg/mL: microgram per milliliter
ATCC 25150: Gram-negative
ATCC 6538: Gram-positive
BET: Brunauer–Emmett–Teller analysis
COD: Crystallographic Open Database
CVD: chemical vapor deposition
DI: deionized Water
DMEM: Dulbecco’s modified Eagle medium
DMSO: dimethyl sulfoxide
DNA: deoxyribonucleic Acid
EDS: energy-dispersive spectroscopy
FBS: fetal bovine serum
h: hour(s)
HaCaT: healthy human skin cell line (ATCC HB-241TM)
HDFs: human dermal fibroblast cells
Hep3B: hepatocellular carcinoma cell line (HB-8065, ATCC)
HhDPCs: human hair dermal papilla cells
HRTEM: high-resolution transmission electron microscopy
HUVECs: human umbilical cord endothelial cells
LB: lysogeny broth
MBC: minimum bactericidal concentration
MCF10A: healthy breast cells (CRL-10317, ATCC)
MCF7: breast cancer cell (HTB-22, ATCC)
MDA-MB-231: breast cancer cell (HTB-26, ATCC)
mg/mL: milligrams per milliliter
MIC: minimum inhibitory concentration
min: minute(s)
ml: milliliter
m^2^/g: square meters per gram
MoB_2_: molybdenum diboride
MoCl_5_: molybdenum pentachloride
MoO_*x*_: molybdenum oxide
MoS_2_: molybdenum sulfide
MTS: (3-(4,5-dimethylthiazol-2-yl)-5-(3-carboxymethoxyphenyl)-2-(4-sulfophenyl)-2H-tetrazolium)
nm: nanometer
NMs: nanomaterials
NPs: nanoparticles
PBS: phosphate buffer solution
PSA: penicillin/streptomycin/amphotericin
PSD: particle size distribution
PVD: physical vapor deposition
ROS: reactive oxygen species
SAED: selected area electron diffraction
SEM: scanning electron microscopy
SSR: solid-state reaction
XPS: X-ray photoelectron spectroscopy
XRD: X-ray diffraction spectroscopy
