# Supplementary material for: Molybdenum Diboride (MoB2) Nanoparticles via a Facile Molten Salt Route: Synthesis, Characterization, Cytotoxicity, and Antibacterial Studies
Source: ACS Appl Bio Mater. 2025 Jul 14;8(8):6745–54. doi: 10.1021/acsabm.5c00161 (PMC12365880; doi:10.1021/acsabm.5c00161)
Supplement: Supplementary file 1 [file mt5c00161_si_001.pdf]

SUPPORTING INFORMATION

Molybdenum diboride (MoB_2) nanoparticles via a facile molten salt route: Synthesis, characterization, cytotoxicity and antibacterial studies

Hamide Aydın [a, b], Burcu Üstün [c], Utkan Şahintürk [d, e], Nurdan Sena Değirmenci [f], Fikrettin Şahin [f], Sedef Kaptan Usul [g], Ayşe Aslan [g, h], Muslum Demir [b, i], Ümran Kurtan [j*]

[a] Department of Chemistry, İstanbul University-Cerrahpaşa, 34500, İstanbul, Türkiye

[b] The Scientific and Technological Research Council of Türkiye (TUBITAK) – Marmara Research Center (MAM), Materials Institute, 41470, Gebze, Türkiye

[c] Department of Chemical Engineering, İstanbul University-Cerrahpaşa, 34500, İstanbul, Türkiye

[d] Department of Mechanical and Metal Technologies, Vocational School of Technical Sciences, İstanbul University-Cerrahpaşa, 34500, Istanbul, Türkiye

[e] Institute of Nanotechnology and Biotechnology, İstanbul University-Cerrahpaşa, 34500, İstanbul, Türkiye

[f] Department of Genetics and Bioengineering, Faculty of Engineering, Yeditepe University, 34755, İstanbul, Türkiye

[g] Gebze Technical University, Bioengineering Department, 41400, Kocaeli, Türkiye

[h] Gebze Technical University, Institute of Energy Technologies, 41400, Kocaeli, Türkiye

[i] Boğaziçi University, Engineering Faculty, Department of Chemical Engineering, 34342, İstanbul, Türkiye

[j] Department of Materials and Materials Processing Technologies, Vocational School of Technical Sciences, İstanbul University-Cerrahpaşa, 34500, İstanbul, Türkiye

*Corresponding author, E-mail : umran.kurtan@iuc.edu.tr

Characterization

The phase composition of MoB_2 was characterized using powder X-ray diffraction (XRD, Panalytical EMPYREAN). X-ray photoelectron spectroscopy (XPS) data were obtained with a Thermo Scientific K-Alpha. Scanning electron microscopy (SEM, Philips-FEI XL30 ESEM-FEG) was used to capture SEM images, while high-resolution transmission electron microscopy (HRTEM, FEI TALOS F200S TEM 200 kV) was employed for HRTEM images. Pore structures were analyzed using N_2 adsorption and desorption isotherms at 76 K with a TriStar II.

Table S1. XRD data.

	2-theta (°)							
	**001**	**100**	**101**	**002**	**110**	**111**	**200**	**201**
**MoB_2-4**	29.65°	34.51°	45.73°	60.80°	-	69.20°	71.64°	79.33°
**MoB_2-8**	29.66°	34.63°	45.74°	60.77°	61.96°	69.14°	71.65°	79.48°
**MoB_2-16**	29.59°	34.63°	45.70°	60.76°	61.75°	69.85°	71.64°	79.50°

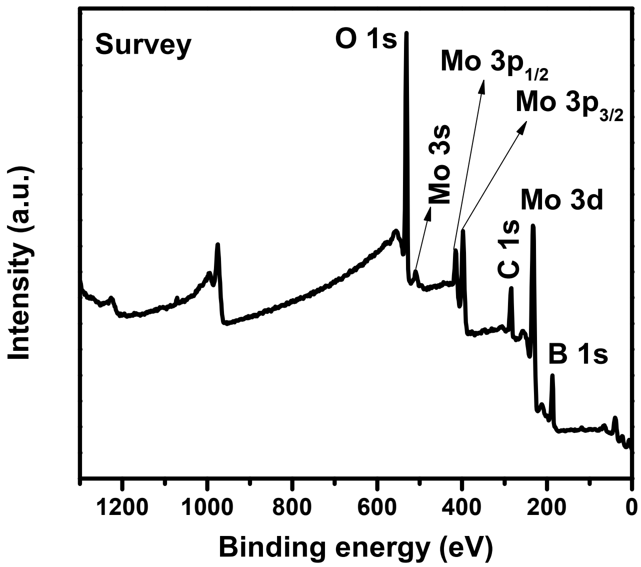

Fig. S1. XPS survey spectra of MoB_2-8.

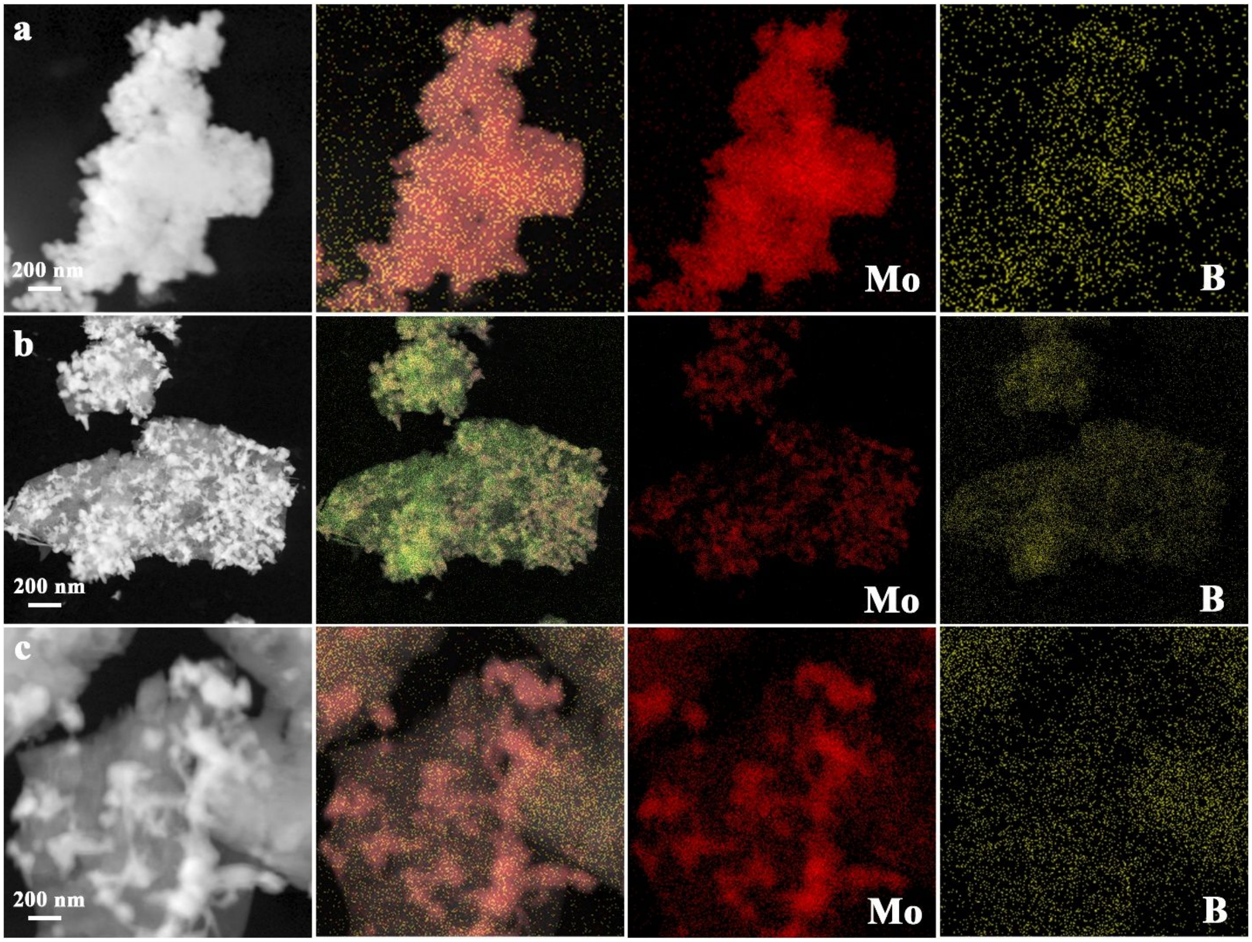

Fig. S2. TEM elemental mapping of (a) MoB_2-4, (b) MoB_2-8, and (c) MoB_2-16.

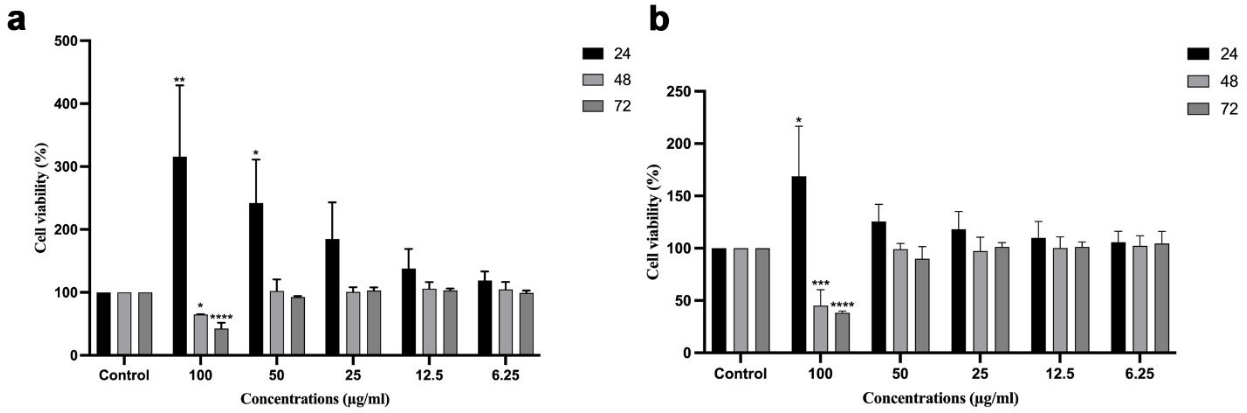

Fig. S3. Effect of **(a)** MoB_2-4 and **(b)** MoB_2-8 treatment on the viability of HaCat cell line. 6.25–100 µg of **(a)** MoB_4 and **(b)** MoB_8 were treated on HaCat cells at 24, 48 and 72 h. MTS assay was performed on the consecutive days of treatment. Absorbance was measured at 490 nm by using a microplate reader. Data shows the average of experiments repeated three times ± SD (****$P \leq 0.0001$, ***$P \leq 0.001$, *$P \leq 0.05$).
